# The fabric of Post-Western sociology: ecologies of knowledge beyond the “East” and the “West”

**DOI:** 10.1186/s40711-021-00144-z

**Published:** 2021-04-19

**Authors:** Laurence Roulleau-Berger

**Affiliations:** French National Centre for Scientific Research, ENS Lyon, TRIANGLE, Lyon, France

**Keywords:** Sociology of knowledge, Cosmopolitanism, Orientalism, Epistemology, Post-colonial studies, De-westernization, easternization

## Abstract

For several centuries, the history of the West has merged with the history of the world. The global economy of knowledge is structured around epistemic inequalities, hegemonies, and dominations. A clear division of scientific practices has developed among academic “peripheries,” “semi-peripheries,” and “core.” The question of epistemic injustice, which includes the indigenization of knowledge, was posed very early in the twentieth century in China, Japan, and Korea without being linked to coloniality, which was the case in Indian sociology. Based on the production of an epistemology shared with Chinese sociologists, we proposed a Post-Western sociology to enable a dialogue—on a level footing—addressing common concepts. This sociology also addresses concepts situated in European and Asian theories that consider the modes of creating continuities and discontinuities as well as the conjunctions and disjunctions between the knowledge spaces situated in different social contexts. We aim to fill the gaps between these social contexts. We will describe an ecology of knowledge in the *Western*-*West*, *the non*-*Western*-*West*, *the semi*-*Western West*, *the Western East*, *the Eastern East*, and *the re*-*Easternized East* situated on an epistemological continuum. While Chinese sociology has constantly oscillated between indigenization and universalism, and while epistemic autonomies are diverse, Chinese sociologists agree that Western sociologies should not be considered hostile to Chinese sociology. We will offer a definition of Post-Western sociology and demonstrate how it can be theoretically and methodologically applied. We will then identify some transnational theories, theoretical discontinuities and continuities, and common knowledge situated in Western and non-Western contexts.

## Introduction

For several centuries, the history of the West has merged with the history of the world. According to Achille Mbembe (2018), the West, which has given the world so much and taken an equal share in return, has become the subject of strong criticism over the past two decades. This critique was initiated by scholars such as Edward Saïd, Dipesh Chakrabarty, Gayatri Chakravorty Spivak, Homi Bhabha, and Syed Farid Alatas. Social science has been profoundly affected by the history of multiple imperialisms, colonialisms, and capitalisms over the last 50 years. The global economy of knowledge is structured around epistemic inequalities, hegemonies, and dominations. A clear division of scientific practices has developed among the “peripheries,” “semi-peripheries,” and “core” with regard to the invisibility of the coloniality of knowledge (Connell 2019). Rajeev Bhargava (2013) challenges the situation in which, in various parts of the world, the analytical categories are derived from the Western experience and argues in favor of putting an end to the epistemic injustice produced by the West.

The question of epistemic injustice, which includes the indigenization of knowledge, was posed very early in the twentieth century in China, Japan, and Korea without being linked to coloniality, which was the case in Indian sociology. Based on the production of an epistemology shared with Chinese sociologists, we proposed a *Post*-*Western sociology* to enable a dialogue—on a level footing—addressing common concepts (Roulleau-Berger 2016; Xie and Roulleau-Berger 2017; Roulleau-Berger and Li 2018). This sociology also addresses concepts situated in European and Asian theories that consider the modes of creating continuities and discontinuities as well as the conjunctions and disjunctions between the knowledge spaces situated in different social contexts (Roulleau-Berger 2016). We will describe an ecology of knowledge in the *Western*-*West*, *the non*-*Western*-*West*, *the semi*-*Western West*, *the Western East*, *the Eastern East*, and *the re*-*Easternized East* situated on an epistemological continuum. We will introduce the idea of the de-multiplication, the complexification, and the hierarchization of new epistemic autonomies vis-à-vis Western hegemonies in sociology and the new epistemic assemblages between European and Asian sociologies. While Chinese sociology has constantly oscillated between indigenization and universalism, and while epistemic autonomies are diverse, Chinese sociologists agree that Western sociologies should not be considered hostile to Chinese sociology. We will discuss several theoretical heritages in Western sociology and their forms of circulation and appropriation in Asia. We will consider Confucian heritage as an Eastern one and examine how it is revisited today in Chinese, Japanese, and Korean sociology. We will offer a definition of Post-Western sociology and demonstrate how it can be theoretically and methodologically applied. We will then identify some transnational theories, theoretical discontinuities and continuities, and common knowledge situated in Western and non-Western contexts.

### From the global turn to the non-Western West

After deconstructing the de-provincialization of European universalism, various theories have been advanced: Dirlik (2007) proposed the theory of global modernity, Ulrich Beck (2000) introduced the theory of cosmopolitanism, and Shmuel Eisenstadt (2000) produced the theory of multiple modernities using a comparative civilizational perspective to describe the plural forms of modernities in diverse historical and structural contexts. However, following in the footsteps of Shmuel Eisenstadt (2000) and Göran Therborn (2003); Beck and Grande (2010) have studied the varieties of modernity and nonmodernity and considered not only how they coexist and challenge each other but also how they are *entangled* with each other in various ways.

More recently, we must consider the construction of *non*-*Western Wests* by certain Western scholars in the social sciences (Santos 2014; Dufoix 2013; Bhambra 2014; Brandel et al. 2018; Koleva 2018; Roulleau-Berger 2011, 2016). Epistemologies of the South have been produced by social scientists based in the global South and the global North to recognize an epistemic, cultural diversity. Epistemic injustice also invites us to consider an epistemology of nonvisible knowledge. This requires “a decolonial break” from Eurocentric epistemologies to consider a plurality of knowledge spaces (Savransky 2017) and what Raman (2017) called “subaltern modernity as a process of epistemological-spatial/temporal/agential-coalescence constituting a transverse solidarity politics” to demonstrate how resisting subjects produce livelihood-environmental resistance. In another closed way, Bonelli and Vicherat Mattar (2017) have proposed the sociology of equivocal connections within a plurality of sensory worlds.

Santos (2014) proposed to develop an *Epistemologies of the South* that concerns the production of ecologies of knowledge anchored in the experiences of resistance in the anti-imperial South. Santos believes that North-centric and Western-centric thinking is abyssal, and he distinguished between abyssal exclusions and non-abyssal exclusions. Santos also considered the return of the colonial and the return of the colonizer and used the term *subaltern insurgent cosmopolitism* to describe the global resistance against abyssal thinking. He invites us to move from an *epistemology of blindness* to *an epistemology of seeing* based on the creation of solidarity as a form of knowledge and the mutual recognition of the Other as equal. Thus, three epistemological approaches are proposed: to produce a new constellation of knowledge and achieve a transformation of mystified conservative common sense into a new emancipatory common sense; to open a space for a noncolonial or decolonial order, i.e., an epistemology of absent knowledge; and to suggest an epistemology of absent agents through a revisitation of the representations and limits of knowledge (Santos 2014). Thus, a definition is required of an anti-imperial South space that embodies a plurality of epistemological Souths structured around counterknowledge born out of struggle. Bhambra (2014) proposed *connected sociologies* in arguing for recognizing historical connections generated by scientific hegemonies, colonialism, dispossession, and appropriation. It means using connected histories to engage in a revision of sociology and the social sciences.

In dialogue with Santos, for Bhambra (Santos and Bhambra, 2017), *connected sociologies* is a means of reconstructing theoretical categories and creating new understandings that incorporate and transform the previous ones. Both Santos and Bhambra ask whether the aim should be toward singular global sociology or several alternative global sociologies. Brandel et al. (2018) propose to unravel the concept of world anthropology and to deconstruct the category of the global South in demonstrating that different regions of the world have not carried the same value for the making of cosmopolitan disciplinary traditions. For these authors, in anthropology and sociology, the theme of American hegemony resonates in different locations in different ways, and they advocate being attentive to the multiple contestations and employments of concepts within transnational circulations, the plurality of traditions and styles of thought, to move behind the clichéd binaries of Eurocentrism and Orientalism. This means producing sociological knowledge in a pluricentric world and asking whether a secular and democratic nation could be built on the concepts and institutions of colonial provenance.

As global entanglement and interconnectedness are the conditions required to understand the assemblages and dis-assemblages between Western and non-Western societies, we can take into account new forms of methodological nationalism from non-Western societies. For example, Manuela Boatcă argues that rethinking Europe based on its Atlantic and Caribbean borders successfully challenges the Western notion of Europeanness and the modern Nation-state. She advances the notion of Europe as a creolized space by taking into account the regional entanglements to which European colonialism and imperialism have given rise since the sixteenth century (Boatcă 2014, 2015). We must also call attention to the subaltern Westerners by invoking social scientists’ work in the “European semi-periphery,” such as those who have witnessed the rise and fall of different political and social regimes and are aware of the arbitrary and contingent nature of societal institutions. Blagojevic (2010) demonstrated that social scientists in European semi-peripheral countries are trapped in a vicious circle of exclusion and marginalization in the centers of international academic life. They are caught in a series of contradictions—described here as the Catch-22 syndrome. To be included in international scientific arenas, they are asked to abandon their original interests, deform their knowledge and original theoretical presuppositions, and distort the definition of their native context by cloning Western theoretical models. In the face of pressure from their peers, they defer to Western centers of science in a context of hierarchical power and resource dependency between strong Western countries, mostly the USA and the UK. Marina Blagojevic wrote, “What is new is that Western parochialism is being re-packaged and reconstructed as a universal science of the social.” In Eastern and Central Europe, Koleva (2018) has developed non-hegemonic sociology in reestablishing continuities with the past of the discipline, and she argues the view of existing unity in the totalitarian experiences of Eastern European sociologies. This unity is due to the “community of shared destinies” formed by Central and Eastern European countries after World War II. She further questions the diversity of knowledge production both within the separate national sociologies and across them. In Central and Eastern Europe, the entire postwar history of sociology in communist countries shows that, despite the hostile conditions in which it was practiced, the scientific tradition of production of knowledge was successfully maintained. After the hibernation of sociology during the first half of the 1950s, Svetla Koleva shows how the restoration of sociology was reorganized in different research fields from the mid-1950s to the late 1960s around three tendencies: a Marxist theoretical perspective; a non-Marxist approach to different theoretical schools and empirical sociology inspired by American sociologies; and an orthodox Marxism. Then, after the Prague Spring, the research fields were reconfigured, and methodological reflection in empirical sociology and a plurality of epistemic autonomies gradually emerged as the Easternization of the Westernized East.

Some scholars open their theoretical spaces in Western countries, separated from Western hegemonic knowledge and breaking the dichotomy between the North and the South, between the West and the non-West, and between modernity and tradition. Thus, in the sense of Tilley (2017), any decolonial knowledge production must involve consideration of a political economy of knowledge. It means a resisting-piratic method by conducting research in new ways and returning our work to the intellectual commons. Tilley has criticized the linear and static conception of time and space in European epistemologies and has suggested that in constructing alternate theories of modernity (Patel 2010), the ethics of method must be scrutinized to take into account diverse indigenous conceptions of time.

### Re-easternization of the Westernized East

In East Asia, the creation of the East Asian Sociologists Network (EASN) in 1992 by Chinese, Japanese, and Korean sociologists to produce connected sociologies represented a major challenge. In their preface to *A Quest for East Asian Sociologies*, (Kim, Li, Yasawa 2014), Kim Seung Kuk, Li Peilin, and Shujiro Yazawa affirm that the EASN was an initiative to construct a new East Asia and radically reflexive sociology calling into question the concept of Western modernity. Today, in Asian regional forums, intellectuals from China, Korea, and Japan continually discuss the modes of producing epistemic autonomies. In this regard, we do not pretend to present the main trends in Japanese, Korean, and Indian sociology but rather seek to mobilize certain sociologists who play an important role in these forums.

#### Epistemic autonomy between Western theory and Korean reality

According to Park and Chang (1999), Western sociology was introduced in the early twentieth century in a specific historical context during which Japanese colonialism, the Korean War, and the Cold War had a strong influence on Korean sociology. From 1910 to 1920, Korean sociology was suppressed under Japanese colonialism, leading to a gap in Korean intellectual history; in the 1930s, Korean intellectuals turned to Marxism to criticize Japanese imperialism. Between 1946 and 1996, Korean sociology had a history of constant exposure, accommodation, and criticism with regard to Western sociology and the issues of universalism/particularism. While American sociology was very influential from 1953 to 1970, in the 1970s, Korean sociologists reintroduced Western hegemony and the indigenization of knowledge. Faced with the singularity of the Korean experience characterized by economic growth, individualization, and democratization, the 1990s saw a fundamental change in the relationship between Korean sociology and Western thought. Figures such as Shin Yong-Ha invited sociologists to rebuild a theory that accounted for Korean history and addressed factors such as Japanese colonialism and North-South national division.

After the 1990s, the necessity of an epistemic autonomy affirmed itself rather early in South Korea. In political science, Kang (2006) analyzed the Korean academic dependence on American political science, which led to the marginalization of the Korean experience through Western ethnocentrism. Shin Kwang-Yeong (2013) identified three modes of hegemonic social sciences constructed in double indigenization of social sciences: the development of paradigms in the West, the dominance of the located concepts and theories associated with institutional power, and the contested hegemony in terms of unavailable alternative theories. Kim (2014) spoke of an *East Asian Community* (EAC) and introduced the idea of the invention of an *East Asianism* to propose the orientalization of an East Asia westernized from hybridizations of “Western” and “non-Western” knowledge and to move toward a cosmopolitan society by constructing transnational regional identities. In an in-depth dialogue with Han and Shim (2010) also supported a “bottom-up” methodological cosmopolitanism by putting forth East Asian identity, history, and culture to get over the “risk society” and develop the idea of reflexive modernization. Thus, a re-appropriation of Confucianism is required when developing this new vision as an alternative to the Western theory of hegemonic instrumental rationality (Han 2019). Taking the same perspective, Chang (2010, 2017b) developed the theory of compressed modernity and internalized reflexive cosmopolitization: “compressed modernity as a civilizational condition in which economic, political, social and/or cultural changes occur in an extremely condensed manner in respect to both time and space, and in which the dynamic coexistence of mutually disparate historical and social elements leads to the construction of a highly complex and fluid social system.” He explained the extreme changes, rigidities, complexities, intensities, and imbalances in South Korean and Asian societies. Chang (2017a) extended Shmuel Eisenstadt’s thesis and Göran Therborn’s thesis of the internal multiplicity of modernities across varying units or agencies of modernity in each national society in distinguishing *colonial dialectical modernity*, *post*-*colonial reflexive institutionalist modernization*, *post*-*colonial neo*-*traditionalist modernity*, *cosmopolitan modernity*, and *subaltern liberal modernity*. Chang Kyung-Sup analyzed the variations of compressed modernity and internalized reflexive cosmopolitization in advanced capitalist societies, transition societies, and underdeveloped societies.

#### Unstable epistemic autonomy in Japan

In Japanese sociology, the development of an epistemic autonomy is being constructed in different terms. Yatabe (2015) recalls that, for 150 years, Japanese intellectual history was organized around a double process of pendulum oscillation between passion for the West and exaltation of the Japanese and Asian spirit, on the one hand, and for going beyond modernity, on the other hand. According to Yasawa (2014), Japanese sociology has been developed by a partial modification and arrangement of Western sociology. After the war, the influence of American positivism and Talcott Parsons became even stronger, and Japanese sociologists were confronted with the theory of civil society and Marxism. However, after the rapid economic growth of the 1960s and the 1980s, the united influence of theorists such as Jürgen Habermas, Anthony Giddens, Ulrich Beck, and Pierre Bourdieu increased. Simultaneously, the theory of Yasuma Takada and Kunio Yanagita was re-evaluated, and Ariga’s rural sociology and Suzuki’s regional urban sociology became new sociology classics. There was a tendency to create endogenous sociologies based on various traditions of Japanese humanity and social science. Kazuko Tsurumi’s theory of endogenous development is a typical example of that approach. Shujiro Yazawa explains that, after 1980, Post-Modern Japanese sociology was developed, and an indigenous sociological theory began to form, especially the critical theory of Takeshi, Hiroyuki Torigoe in environmental sociology, and Tsuyoshi Hoshikawa in the 2000s in terms of public space.

In Japanese sociology, the theory of individualization rallied many sociology scholars (Elliott et al. 2013). Yamazaki (1984) distinguished the “hard individualism” in industrial society, the “soft individualism” in postindustrial society, and the “individualization of misfortune” during the decline of the Nation-State. In a postwar context, Keishi Saeki (1997) defined individualization as a process of emancipation from local and traditional communities. Katagiri (2013) described three selves in different periods of Japanese society: the individualized self, the private self, and the psychological self. Yasawa (2014) showed how reflexive sociology organized around the production of a transcendental subject is developing, resulting in a post-reflexive self, a hermeneutic self, and a pre-reflexive self.

In this unstable epistemic autonomy, the Great East Japan Earthquake and tsunami have changed the vision of sociology and anthropology by redefining their status in the public and political space. Today, the sociology of social movements, especially anti-nuclear movements, has taken a central role in Japanese sociology. Yasawa (2019) is developing a theory of risk culture instead of risk society, which was originally presented by Mary Douglass and Scott Lash—following the East Japan Great Earthquake and tsunami disaster to understand noninstitutional, emergent associations, horizontal orders, and reflexive communities. Yama (2012) demonstrated how disaster-affected communities with collective traumatic memories could move toward recovery, putting forward the concept of symbolic recovery. Hasegawa (2015, 2018) introduced the problems related to the continuities and discontinuities of political activism in the sociology of social movements following the Fukushima disaster. Nomiya (2019) opened a theoretical perspective on social movements as “networks of meanings” by creating a mental map from Hiroshima to Fukushima to understand the change in mentality. Takakura (2016) suggested that disaster salvage anthropology should be conducted among many stakeholders (anthropologists, government, NGOs, and the people affected) from a perspective of intangible cultural heritage.

#### Coloniality and indigenization in India

In India, social sciences were born during the English colonial period, with sociology emerging in 1919 at the University of Bombay. In the sociological tradition, Sujata Patel defined two broad epistemes: colonial modernity and methodological nationalism. Govind Sadashiv Ghurye, the father of Indian sociology, developed an orientalist and Indological approach to understanding Indian society by focusing on the continuities of traditions in social practices and institutions in modern India (Patel 2010). Madan (2011) explains the history of the construction of the discipline—a methodological nationalism constituted an intellectual and political resource in India to distinguish itself from the dominant colonial knowledge and to develop an alternative voice. Notably, in Calcutta in the 1920s and the 1930s, Lucknow and Mumbai, the only universities where sociology and anthropology were taught, saw the appearance of radical nationalisms that encouraged the studies on the poverty of peasants, artisans, and marginal groups. According to Sujata Patel, in the Lucknow School, being Indian was defined simultaneously considering modernity and the traditional/local/indigeneity. According to T. N. Madan, Radhakamal Mukherjee developed a rather universalist perspective, and Dhurjati Prasad Mukerji, a culturalist perspective, to construct social science categories. At the beginning of the 1970s, M. N. Srinivas, Chair of the Department of Sociology at the University of Delhi, proposed social anthropology, or rather situated sociology, based on the study of communities and castes from the posture of an “insider” in Indian society. From Srinivas’s perspective, the discourse of colonial modernity was paroxistic. During the post-independence period, sociology produced a replica of anthropological theories and struggles to combat the production of a colonial state discourse on the Indian, or “Sinhalese,” society as a non-modern society (Madan 2011). A.R. Desai, a Marxist, criticized Indian nationalism by developing perspectives on nation and class in the colonial period and studying new social stratification in rural India.

Today, some Indian intellectuals support the idea of deconstructing the provincialism of European universalisms by recognizing a genealogy of knowledge that is both European and linked to colonial history. Some Indian sociologists are rethinking earlier themes such as marriage, family, caste, religion (religious rights, pogroms against Muslims, and other minorities) and are reframing these subjects by way of areas such as intersectionality, exclusions, identities, and queer positions (Patel 2017). Sujata Patel wrote, “the heritages of the epistemes of colonial modernity and methodological nationalism entangled our scientific practices and professional cultures in non-modern practices and legitimized traditionalist perceptions and practices” (Patel 2017, p. 143). We can see how a form of epistemic autonomy was developed in India based on methodological nationalism.

Materially, these forms of epistemic autonomies come together in networks of forums, of colloquia. In China, Japan, Korea, and India, different forms of *cosmopolitan imagination* (Cicchelli 2018) are developing, translating differences and diversities of traditions and cultural influences. Political, historical, social, and economic contexts affect the production of the intellectual epistemic autonomies that defend positions, sensibilities, and relationships to different worlds in the scientific field and depend on margins of action and liberty that vary from one country to another.

### Chinese sociology between indigenization and universalism

In the context of scientific globalization, the presence of changing hierarchical hegemonies, the confirmation of the invisibility of some academic spaces, and even the marginalization of some other academic spaces bring the issue of the indigenization of scientific practices back into the spotlight. The issue of the production conditions of the sinicization of social sciences lies at the heart of the *bentuhua* (本土化), the indigenization of sociological knowledge. Additionally, the historical trajectory and fluctuation of sinicizing sociology in China’s early periods reflect the relationship between Chinese and Western sociology (He 2018b).

In China, the issue of epistemic autonomy vis-à-vis Western sociology was first raised in the 1930s by Sun Benwen (Wen and Wang 2012). Indeed, within a short range from historical materialism, he developed a sociology of the individual that played a decisive role in the process of sinicization of Chinese sociology. Epistemic autonomy can be observed from the moment Sun Benwen, president of the Chinese Society of Sociology, launched the movement to indigenize sociology to produce independent thinking while mobilizing Western methods and theories. At the time of the discipline’s renewal in 1979, the issue of the sinicization of the discipline resurfaced, in particular, whether it should be rebuilt *next to*, *with*, or *against* Western thought by affirming the refusal of hegemonic postures and seeking benchmarks in past or present times of Chinese civilization as well as in filiations, displacements, and hybridizations with European and American ideas (Roulleau-Berger 2008). The question of the epistemic autonomy of sociology through its sinicization has always existed in the idea of producing sociological knowledge emancipated from the hegemonic thought related to “Western” sociologies. Thus, contemporary Chinese sociologies appear to find themselves in a partially non-Western mosaic of contextualized and revised constructivisms that are linked to historical and civilizational contexts. In Chinese sociology, epistemic autonomies develop in different theoretical perspectives and show a real internationalization of the discipline and the consolidation of new borders.

In 2019, the publication of two articles written by Xie (2018) and Zhou (2020) sparked controversy and stirred a passionate debate in Chinese sociology. Indeed, this allowed for the questioning of American hegemony, which imposes standards of international scientific legitimacy such as the necessity to publish in the *American Journal of Sociology* as a supra-norm of scientific recognition.

According to Xie Yu, who considers the indigenization of sociology a nonissue, Chinese researchers often use the concept of indigenization with three different meanings:

1. A spatial and temporal contextualization of concepts that appear to be specifically linked to Chinese society, such as the *chaxu geju* (差序格局), a differential mode of association among individuals (Fei 1992).
2. The use of Western theories and sociological methods and their reinterpretation in local situations in China.
3. Some researchers believe that Chinese sociology must develop new theories and methods based on traditional culture (such as Confucian ethics, the *chaxu geju*, and the traditional Chinese cognitive system), and even form a new paradigm rooted in Chinese history and culture at the epistemological level. Indigenization means “rethinking and criticizing foreign systems of sociological knowledge.”

Zhou (2020) defines the indigenization of sociology as the involving issue of a Western hegemonic power in the output of the discipline and as taking differentiated shapes in different historical periods. For instance, when the Chinese Society of Sociology was founded in 1930 by Sun Benwen, Xu Shilian, Wu Jingchao, Chen Da, Tao Menghe, Pan Quentin, You Jiade, and Qian Zhenya, the main issue was integrating Western sociology and testing it within Chinese society to create a sociological theory through the creation of the Synthesis School (He 2018a).

For instance, at the time of the re-foundation of the discipline in 1979, the issue of the sinicization of sociology was not explored in the same terms as it was urgent to import and integrate all the Western theories before reinterpreting them, refusing them, or synthesizing them with pre-1949 Chinese sociologies. Today, it would instead be a matter of the reinvention of hybrid paradigms in which the approaches related to Chinese thinking occupy a central position. For Zhou Xiaohong, the main issue is the universal value of sociological knowledge beyond the borders within which it is produced. With a close perspective, Liu and Wang (2017) proposed controlled sinicization of Chinese sociology, going beyond the opposition between the universalism of sociology and indigenization as passive resistance to scientific colonialism. Although Chinese sociology, from the perspective of those authors, remains influenced by Marxism-Leninism, by traditional Chinese culture and Western rationalism, they still suggest a focus on how society is constructed, consideration of public life, and the mobilization of a historical and civilizational perspective.

Chinese sociologists agree with the idea that Western sociologies should not be considered antagonistic to Chinese sociology. Unlike Post-Colonial Studies, which invite a de-westernization of colonial knowledge, Western sociologies and Chinese sociology are not analyzed in a mutually exclusionary relationship. Nevertheless, between 1920 and 1952, several sociological schools already existed, and they held different conceptions of sinicization (Li et al. 2009): in the Synthesis School, Sun Benwen studied the foundations of individual behaviors and the social environment from the standpoint of a sociology of the individual (Sun 1935). In the Rural Sociology School, Yang (1930) imported the notion of community in Chinese studies to develop the idea of a common local society by going back to the historical foundations of the tradition of an autonomous rural Chinese society. Pan (1931) revisited the Western evolutionist theory and proposed a new reflection on the practices and rites of the Chinese tradition to overcome the East/West dichotomy.

At the time of the re-foundation of sociology in 1980, the issue of sinicization was strongly debated in the theoretical framework of two different traditions: indigenization through Hong Kong and Taiwanese sociologies or indigenization through the reconstruction of the discipline in the Chinese mainland. Then, starting from the year 2000, the main wave of the sinicization of sociology defined itself in contrast with hegemonic Western thought and excessive indigenization to find a strong place in the international academic environment. For instance, Liu and Su (2019) argue that social and cultural practices in China are in part universal, and they refuse the idea of an exaggeration of the Chinese exception.

### Diversification of epistemic autonomies in China

If Chinese sociology developed epistemic autonomy without facing the issue of the relationship between the colonist and the colonized before 1949 and since 1979, modern Post-Colonial sociology has witnessed the development of a multitude of epistemic autonomies from different theoretical perspectives, thus demonstrating a real internationalization of the discipline and the consolidation of new boundaries. Today, many Chinese sociologists draw from the history of Chinese thought the intellectual resources as necessary for the reinvention of sociology that can be fed by and emancipated from Western influences.

The renaissance of sociological thought in China represented a fundamental moment in the history of global thought (Li et al. 2008). There was a type of epistemological, ethical, and political indecency in Western worlds that ignored the sciences of Chinese society, which constitute a practice as ancient as that of the West (Roulleau-Berger, Guo, Li and Liu, 2008). Although the Chinese language could form a barrier, it was above all the orientalisms that fixed the frontiers of perceived and lived knowledge, which are represented as more legitimate than other knowledge. Today, in Chinese sociology, we can distinguish four forms of epistemic autonomy: *historic epistemic autonomy*, *alternative epistemic autonomy*, *local epistemic autonomy*, and *plural epistemic autonomy*.

A *historic epistemic autonomy* refers to the re-establishment of continuities with epistemic frameworks constructed before 1949 and then forgotten. In Europe, most intellectuals ignore renowned pre-1949 Chinese sociology. In *A History of Sociology in China in the First Half of the Twentieth Century*, (Li et al. 2009; Li and Qu 2011, 2016) demonstrated how Chinese sociology flourished in a context of intellectual blooming comparable to that of the spring and autumn periods and to that of the warring States. This is a context of social reform in which intellectuals defend pragmatic positions. Li Peilin and Qu Jingdong note that in his 1923 conference “A history of thought in China during the last three centuries,” Liang Qichao considered the idea that Chinese thought, since the sixteenth century, has been a pragmatic one that has developed in reaction to 600 years of Taoism. In the scientific history of Chinese sociology in the first half of the twentieth century, Li Peilin and Qu Jingdong distinguish five currents of ideas: historical materialism, rural construction, and the social survey campaign, the “Chinese School,” the “academic school,” or “scholastic school,” and the study of social history. The “social survey movement” corresponds to an important movement hatched at the beginning of the twentieth century. Qu (2017a) considers, “The inspiration of classic sociology made us realize that the structure and transitions of modern Western society have different traditions, structural conditions, and senses of real-life experiences. The evolutionary trend of history is by no means purely unified. The modernity we see today is one of the possibilities that was derived from modernity; furthermore, different civilizations and communities make their adjustments, transformations, and reconstructions between tradition and modernity.”

The production of an *alternative epistemic autonomy* could be represented by He (2018a). He has used the concept of the captive mind from Alatas (1974) to analyze the lack of autonomous social sciences in Asia and to explain the monopoly of social sciences that remains intact in a context of abandoning Eurocentrism in the social sciences. He considers whether the tense relationship between the West and Post-Colonial sociology cannot be found in the Chinese context and whether Chinese sociology has produced historical materialism as its own counterpart. In the continuity of a long history of sinicization, Chinese sociology could produce an epistemic autonomy by walking through the “post-colonial fog.” He Yijin proposed the notion of alternative autonomy in analyzing the self-adaptations of Chinese sociology in the 1950s. Taking a novel approach, he shows how Chinese intellectuals have remained relatively silent in the debate about Post-Colonial Studies. The production of an alternative epistemic autonomy means taking into account the past, the present, and the future of Chinese sociology; thus, revisiting the historical trajectory of sociology is an expression of an intellectual voice. He Yijin analyses how, on the one hand, the sinicization of sociology has been proposed by native scholars to indigenize Western sociology and, on the other hand, unlike post-colonial sociology, which treats Western sociology as an object to debunk or overthrow, Chinese sociology has absorbed Western sociology in producing hybridizations, reformulations, and readjustments to Chinese society. The real challenge is how Western sociology can be sinicized and, conversely, how Chinese interpretations of Western sociology have been changed.

By affirming a *local epistemic autonomy*, we will mobilize the theory of oriental modernization by Li (2015) and the sociology of action by Zhou Feizhou. Li Peilin largely introduced the concept of oriental modernization to open a space where theoretical thinking can occur in a cohesive and not separate manner, as has often been the case between Chinese society and modernization. Oriental modernization is an important topic for Post-Western sociology; oriental modernization is proposed because “oriental society” had almost no relationship with modernization in the past several centuries. It has seemed to many theorists that the possibility of oriental modernization can only be a suspension. Thus, oriental modernization is not exactly a concept of regional regulation; it should include all developmental paths of new experiences in providing world modernization that is different from those of the West (Li 2015). We can also illustrate *local epistemic autonomy* with Zhou (2018), who advanced the sociology of action based on a society of *guanxi* (关系interpersonal relationships). These notions describe traditional Chinese social structure and its organizing principles that rely on *chaxu geju* (the differential mode of association), or the familial and personal ties that extend to political, economic, and social relationships. He distinguishes Western society, which is characterized by a social structure based on the production of individuals, from Chinese society, which is structured on the reproduction of family relationships.

Finally, Xie Lizhong defined *hou shehuixue* (后社会学Post-sociology) as pluralistic discourse analysis. We cannot believe that all the controversy in social research was based only on certain discursive disputes but that all social realities constructed by people having their own objectivities and that social phenomena have no essence. Post-sociology is a new perspective in China based on the abandoning of “given realism,” “representationalism,” “essentialism,” and “fundamentalism,” and on the promotion of a multidimensional, dynamic, processual, and plural space for sociology (Xie 2012a, 2012b). Post-sociology is opening an epistemological space based on a plurality of theoretical paradigms, a Chinese way to establish theoretical assemblages, conjunctions, and continuities beyond Western and non-Western theory and to produce Post-Western sociology. From a Post-sociology perspective, Xie (2020) developed a theory of discursive pluralism, distinct from geographic pluralism, to describe Western and non-Western societies beyond epistemic borders linked to indigenous sociological theories. While geographic pluralism favors the indigenization of sociological thought by producing a discourse in a specific time and space, discursive pluralism appears to be linked to a form of universalism containing a wide range of discourses that may be applied to any time and space. Moreover, geographic pluralism includes the idea that sociological discourses in non-Western societies cannot be used to understand Western societies. Here, we can discuss *plural epistemic autonomy*, which will become foundational for the construction of Post-Western sociology.

### Legacies and the sociological imagination

In this section, we will first discuss the multiple heritages of Western sociology in Post-Western sociology and their forms of appropriation in Asia, notably Marxism, the theory of Durkheim and Weber, the Chicago School, and phenomenological theory. We will then examine how Confucian heritage is revisited in Chinese, Japanese, and Korean thought through sociological imagination.

#### Western heritages and transformation

##### Marxism as sociological tradition

In China, where Marxism was and still is very influential, Sun Liping, Guo Yuhua, and Shen Yuan (Shen and Guo 2010) from Tsinghua University formulated the sociology of transformation based on the sociology of practice inspired by a Marxist perspective in the relationship between the upper-class and the working class. They suggested that Karl Marx was concerned with the formation of classes and class conflicts under capitalism, Max Weber theorized the cultural foundations of capitalism, and Emile Durkheim focused on the capitalistic division of labor. In this way, they emphasized that communism, such as capitalism, is a way of life—a civilizing process that exists in everyday practices. Sun Liping, Guo Yuhua, and Shen Yuan borrowed from Pierre Bourdieu’s sociology of practice by focusing on processes and practices rather than structures. For Li Lulu (2008), he has remained more faithful to Pierre Bourdieu and Marx by focusing on the phenomena of the reproduction of social inequalities in China.

In 1945 in Korea, the post-liberation era saw disputes emerge between Marxists and non-Marxists, with Marxist narratives spreading throughout Korean society to fight against Japanese coloniality. Marxism was recognized as one of the great sociological traditions. From 1980 to 1990, young scholars considered Marxism to be a viable alternative in the social sciences to analyze Korean capitalist society (Park and Chang  1999).

In India, A.R. Desai mobilized Marxist theory to consider social classes, the nation, and the state in a radical Indian nationalist language. As Chair of the Department of Sociology at the University of Mumbai, A. R. Desai was considered by D. N. Dhanagare to have produced a real body of critical thought emancipated from Western Marxist sociologies.

##### Durkheim’s and Weber’s theory in China

Durkheim’s theory was introduced very early in China. In 1925, after studying in France, Xu Deheng translated *The Rules of Sociological Method*. He played a major role in translating French works; however, he adopted a new orientation and became interested in Marxist theories. In 1930, Cui Zaiyang translated *Moral Education*, and Wang Li translated the *Division of Social Work*. Throughout this period, several theses were supported on French sociology: that of Xie Kang in 1931; that of Xie Zhengru, which the same year situated the theories of Durkheim in social psychology; and that of Wu Wenzhao in 1932. During this period, Yang Kun (1932a, 1932b) played a decisive role in spreading French sociology in China and structuring the sociology of religion. Alongside Yang Kun, other Chinese sociologists, including Cui Zaiyang, Hu Jianmin, Ye Fawu, studied in France and fueled the debate on the Durkheimian theory in China between 1925 and 1934 (Roulleau-Berger and Liu Zhengai 2012b.

Today, intellectual legacies and specific theoretical approaches are still intertwined in Chinese sociology. Durkheim’s theories were developed for the first time in 1949. They were taken up twice after 1979, and they have since been revised, for example, by Wang Hejian, Chen Tao, Qu Jingdong, and Zhao Liwei. For example, Wang (2005) proposed to revisit Durkheim by taking an interest in producing moral goods in the modern Chinese economy. From the perspective of economic sociology, this involves linking economic morality and the social structure, based on an analysis of professional relationships. Chen (2013) is also interested in the contractual society described by Durkheim where morality is generated by communal life and where the autonomy and freedom of individuals, constrained to social facts, are based on the respect of social rules to produce a “normal” society. For Qu (2017b), in Durkheim’s perspective, collective life transcends individual existence. If studying the moral and social order requires starting from the production of norms in everyday life, the relationship between society and state cannot be thought of as antagonistic but rather as a continuum in which the ethics of professional groups and civic morality link individuals to the state. Last, for Zhao (2014), Durkheim’s theory of suicide appears to be a major contribution that remains very important to understanding modern societies. In this sense, one could say that Durkheim’s study of suicide as a general social fact explains the modern human condition.

Weber’s theory also had a very strong influence in China. His books on China, *Confucianism and Taoism*, and *The Religion of China*, occupied a prominent position in Weber’s research system. First, Weber’s China studies were regarded as Eurocentric and hegemonic studies. According to He (2020), Weber’s theoretical ambition was to understand the various types of rationalism in the world’s civilization systems, indicating that the world’s civilization systems are a rational regulatory system of equality, juxtaposition, and coexistence. She asserted that Max Weber overcame the cleavages between western and nonwestern civilizations. His research on China is part of the grand system of the economic ethics of the world’s religions. It is by no means a single process with the West as the only model and teleological orientation; however, it should follow the inherent logic of each civilization system and have its own characteristics. It can be said that this research strategy of his method avoids Eurocentrism. For He Rong, *Confucianism and Taoism* is not only a study of the spiritual temperament of Confucian rationalism, that is, in a dialogue with protestant ethics, but more than half of the work is devoted to the topic of sociology.

##### Chicago School’s heritage

If the localized approach of European urban sociology developed from the 80s could be privileged as in the “community studies” linked to the Anglo-Saxon tradition, we can observe the same movement in Chinese urban sociology since the 80s linked to the influence of Fei Xiaotong. It should also be remembered that the representatives of the Chicago School, Robert Park and his son-in-law Robert Redfield, came to teach sociology in China in 1931–1932 and 1948. In the process of re-foundation of Chinese sociology, Fei Xiaotong was strongly inspired by them. The Chicago School is one of the most important scientific communities in the history of sociology. It has played a major role in the history of Western sociology; however, it has also had a continuous influence in non-Western countries, especially in China. Urban sociology and what Zhou (2004) identified as social psychology, two major fields in the Chicago school, greatly influenced the first generation of Chinese sociologists. The remobilization of the Chicago School in China in the twentieth century is very important and produced *shequ yanjiu* (社区研究community studies).

##### Phenomenological theory

In Japan, the phenomenological theory was highly influential in the 1990s. In China, however, it is quite a recent phenomenon led by young scholars. For example, to solve the basic question of how we can understand each other, Sun (2013) first referred to the prominent Chinese scholars of Fei Xiaotong’s generation. Like Japanese sociologists (Nishihara 2013), he used Schütz’s theory, choosing the concept of the lifeworld based on the we-relationship and intersubjectivity. This approach is connected to Chinese and Japanese traditions. While the concept of subjectivity is barely developed in Chinese sociology, intersubjectivity is dealt with in terms of sociologies inspired by pragmatism and interactionism. However, intersubjectivity is also connected to *guanxi*, a very strong concept throughout the entire Chinese sociological field—and Sun Feiyu gives a Western sense to this Chinese concept. While Sun (2018) posture is highly inscribed in Chinese intellectual tradition from a new perspective of the sociology of knowledge, he explores rationalization in sociology as a modern phenomenon of knowledge and its internal dilemma in the development of social science in China. In European theory, most sociologists do not combine psychoanalysis and social theory; therefore, this approach appears to be very fruitful in defining the boundaries of Post-Western sociology.

#### Confucian legacy, re-actualization, and reinterpretation

In China, Korea, and Japan, Confucianism is a common heritage and is revisited in different ways. In Korean sociology, Confucian heritage and Western sociology are combined; however, in Chinese and Japanese sociology, Confucian heritage is embedded in the Chinese or Japanese traditions.

In Korean sociology, Han (2019) defended a post-Confucian approach—in which there is a balance between individuality and social relations—in reflexive modernity by bringing community back to human rights in an age of global risk society. This author establishes the link between Confucianism as an Asian tradition and reflexive modernity—sourced from Ulrich Beck’s concept of conceiving individuality, community solidarity, and “communal autonomy” in a hybrid way—to understand the agency of social change and to build a kind of cosmopolitan Confucianism in the development of public citizens in risk societies. Han Sang-Jin wrote, “the ultimate goal of the post-Confucian participatory approach to community-oriented human rights lies in the possibility of “unconstrained overlapping consensus through an inter-cultural discourse between the East and the West” (2019, p. 46). Finally, he opened dialogues with Anthony Giddens, Jürgen Habermas, Scott Lash, and Ulrich Beck, particularly on the subject of what makes modernity and the definition of cosmopolitanism. Thus, reappropriation is required in working out this new vision of Confucianism as an alternative to the Western theory of hegemonic instrumental rationality (Han 2019).

In China, Confucian ethics have gradually entered the social sciences in modern Chinese academic history. Among them, the “*guanxi* standard” has become the core of sociological research (Zhai 2020). To revisit Confucianism, Qu (2017a, 2017b) also proposed that a return to historical views and a reconstruction of the sociological imagination to understand the reality of Chinese society should be a return to the theme and context of the modern transformation of Chinese social thought. In a reinterpretation of Kang Youwei’s theory of the Three Eras—an “Era of War”, an “Era of Good Governance”, and an “Era of Peace”—from classics, Qu Jingdong noted that Kang Youwei made an explicit judgment that the modern period was an Era of War, the Idea of Cosmos Unity should be established as the universal value for world history, and the Confucian Religion should be established for the cultivation of mores. Kang Youwei blended Buddhism and Confucianism to initiate changes in the political system by adjusting individuals’ belief structures and sparking changes in customs to trigger institutional changes. Good governance can be brought into cosmic unity in a sinicized religion only through Confucianism. Today, adding Buddhism to his history of the Three Epochs, some social scientists are calling Confucianism a force for transformation in the process of modernizing Asian societies.

In Japanese sociology, Yoshiyuki Yama and other scholars have demonstrated that sociological thought has existed in premodern Japan since the Meiji period. In this period, *Kogaku*, which criticizes the neo-Confucianism of the Edo period, and *Nativism* were born. Post-Confucianism and a sort of sociological thought have their origins in these two approaches. This perspective of Confucianism and Nativism in the Edo Period in Japan has been largely ignored in Western sociology. This author combines the Western sociological theory of ritual by Durkheim, the Japanese Confucianist theory of ritual by Ogyu Sorai (1666–1728), and the narrative theory of Motoori Norinaga “knowing an empathy toward things” to produce a non-Western hybrid or sociology of narration and narrative.

### What is Post-Western sociology?

The publication of Saïd’s work Orientalism (Saïd, 2003) represented a very important moment in the history of post-colonial thought. Orientalism signified the establishment of systems that would intercept, capture, and orient gestures, discourses, and viewpoints. This interception process was particularly selective by making active knowledge invisible and by seizing “inert” knowledge—such as the knowledge associated with ancient philosophies—to incorporate and contain it in subfields. The imperial and post-imperial global economy of knowledge is structured and restructured by power and epistemic injustices. According to Connell (2019), “within the routine practices of hegemonized sociology in the periphery, crucial features of colonization and colonial society are normally overlooked….so we can understand coloniality of knowledge.” Furthermore, we can consider that hegemony is not static and that while “old hegemonies” are disappearing, new hegemonies are emerging.

The post-colonial discourse based itself on the idea of provincializing Europe (Chakrabarty 2000) to consider the “subaltern histories” according to their own value, and it played a key role over the last 30 years in the international debate on the *global turn* and the end of Western hegemonies. However, as Mbembe (2006) stated, post-colonial studies are characterized by their heterogeneity but not their originality; thus, it is not easy to discuss post-colonial theory. However, in the context of post-colonial studies, the “post” does not mean “after” but “beyond” (Kilani 2009)—if the concept of a third space was proposed by Bhabha (2007) to enable heterotopian thoughts to appear—a significant challenge for today’s sociologists is to invest in different types of third space, both situated and globalized, by creating epistemological conjunctions and disjunctions.

According to Patel (2019), Eurocentric knowledge is based on multiple and repeated divisions and oppositions; however, here, Post-Western knowledge is constructed through continuous continuities and discontinuous continuities, discontinuous discontinuities, and continuous discontinuities between located knowledge. Post-Western sociology means working toward displacement and the construction of planes of epistemic equivalence between conjunctive and disjunctive borders of knowledge to struggle against any form of “epistemic injustice,” to quote Bhargava (2013), who considers that there are three forms of epistemic injustice: (1) the imposition of a change affecting the content of epistemic frameworks, (2) the alteration of fundamental epistemic frameworks, and (3) the damaging or loss of the capacity of individuals to maintain or develop their own epistemic frameworks.

How can one define Post-Western sociology? Post-Western sociology does not only mean encouraging a multiplicity of non-Western narrative voices but also, and above all, identifying the theories they contain and identifying how they can assist us in revisiting and reexamining Western theories. Post-Western sociology proceeds from the decentralization and the renewal of a “discursive pluralism” (Xie 2020) originating in *Western West*, *non*-*Western West*, *semi*-*Western West*, *Western East*, *Eastern East*, and *re*-*Easternized East* spaces. Post-Western sociology cannot be conceived according to a binary mode; it is, above all, relational, dialogue-based, and multi-situated. Contrary to global sociology, Post-Western sociology refuses term-for-term structural comparisons and favors intersecting viewpoints concerning registers of understanding, agreement, and disagreement as well as the scientific practices of the co-present actors. Post-Western sociology can also be defined as global critical sociology. However, to progress toward global critical sociology, we have opened intercultural spaces for active dialogue between Western and non-Western sociologies (Roulleau-Berger and Li 2012, 2018).

In Post-Western sociology based on epistemic justice, we are not replacing the North’s hegemony of epistemology with another dominant epistemology; we are producing an ecology of knowledge where diverse forms of knowledge may enter dialogue articulated in cosmovisions and emancipatory practices (Pleyers 2011). We refer to the following ecology of knowledge in *the Western West*, *non*-*Western West*, *semi*-*Western West*, *Western East*, *Eastern East*, and *re*-*Easternized East*. Post-Western sociology is developed in a continuum of assemblages, tensions, and the cross-pollination of different segments of this ecology of knowledge. Through academic and institutional translations, this continuum can be rapidly exposed to forms of coloniality that will vary in intensity and create epistemic hierarchies, as we have shown above.

Post-Western sociology relies on different knowledge processes (Roulleau-Berger 2015):

- “knowledge niches”—which appear to be located in the *Western West*, *non*-*Western West*, *semi*-*Western West*, *Western East*, *Eastern East*, and *re*-*Easternized East*;
- intermediary epistemological processes that encourage the partial transfer of sociological knowledge along the continuum of the *Western West*, *non*-*Western West*, *semi*-*Western West*, *western East*, *Eastern East*, and *re*-*Easternized East*;
- legacies and epistemological spaces in which *Western West*, *non*-*Western West*, *semi*-*Western West*, *Western East*, *Eastern East*, *re*-*Easternized East* knowledge are placed in equivalence.

Post-Western sociology is elaborated from the connections between field practices and the intersecting exploration of what individuals in different situations do, say, and think. It does not utilize the differences but the *intervals* between the perspectives, practices, and concepts of Chinese/Asian and European sociologies to co-produce new knowledge, which is the starting point of the construction process of Post-Western sociology. Thus, it precedes the conception of theoretical and methodological combinations and assemblages. International sociology and global sociology do not imply this erasing of epistemological boundaries: this is precisely where the distinctions among Post-Western Sociology, international sociology, and global sociology lie (Roulleau-Berger and Li, 2018).

How to produce non-hegemonic, Post-Western knowledge? In Post-Western sociology, *doing with or doing together* still appears to be central in the fabric of sociological knowledge in taking into account an alternative political economy of knowledge and *anti*-*piratic mode* (Tilley 2017). Finally, the *cosmopolitan imagination* is necessary to develop sociological studies of cosmopolitanism in different fields and to define the matrices of singularity and the places of plurality. In Post-Western sociology, we will be considering both the local and transnational dimensions of academic research as part of our attempt to analyze the effects of social context on the production of theoretical methodologies based on local research situations. However, we will also be analyzing the transnational flows between the various contexts of knowledge linked to research methodologies and considering both the processes involved in the production of sociological knowledge and cultural variations in research practices. Theories, knowledge, and methods cannot circulate until these equivalences have been established and appear to be relatively stable, and the framework is based on common conventions and norms governing academic research that have been put in place. Practices give rise, in these very different contexts, to sociological knowledge obtained in response to questions that are similar but situated in sociologists own societal experiences; we will be posing questions about the universal value of sociological knowledge.

This signifies the implementation of multi-situated, contextualized tools to account for assemblies and disjunctions between the narratives of societies, which are all legitimate, and to produce a “methodological creolization.” In this instance, here, a Post-Western methodology is federated around dynamic and non-hierarchical combinations of societal contexts, structural processes, individual and collective actions, and situational orders. The Post-Western conceptual space is relayed by a methodological space in which sociologists conceive a plurality of temporalities, places, contexts, and situations in the construction of tools for field investigation to access the plurality of the narratives of society and the multivocality or polyphony (Roulleau-Berger 2012; Bastide 2015). Post-Western methodology leads to multi-situated sociology and a methodological pluralism that does not necessarily mean fields of investigation in several countries but rather in several differentiated places.

In the world, in the context of globalization, big data production has become a strong scientific and political issue. In China, the quantitative survey in the form of the social survey is presented as very important to understand why differences in scales play a decisive role here. In Chinese sociology, very large quantitative surveys are produced in different places to produce multi-scaled national statistics about China’s transformation in social structure and social development in 30 years. For example, Chen (2017) examined transformations in the social structural conditions in ten dimensions: demographics, family structure, urban structure, regional structure, employment structure, occupational structure, organizational structure, ownership structure, class structure, and income distribution structure. In Europe, we have increasingly measured and compared quantitative and qualitative surveys from a longitudinal perspective, such as the European Panel. The launch of the European Panel in 1994 marked a significant step forward in the French Statistics on the Observance of Household Living Standards. The European Panel has three dimensions: longitudinal follow-up, European comparability, and the approach of several themes from the same source. We also developed multiple methods, analyses, and international comparisons.

However, we are faced with a surge of social, economic, political, ecological, and moral risk situations that transform the lives of individuals and groups in every country in the world. As a result, in sociology, we have had to synthesize and diversify an increasing number of quantitative, qualitative, and anthropological methods to produce and invent complex approaches that grant access to close and familiar territories. These territories are mined and perceived as dangerous and distant. Liu (2008) proposed to return to the space-based sociology developed by Fei Xiaotong in relying on the methodological concept of “spatial contextuality” connected with Andrew Abbott’s sociological theory focusing on the analysis of social life and social actions in a specific time-space context. Liu Neng also considers coming back to space-based sociology to counterbalance the “de-contextualization error” of modern quantitative sociology. To define “spatial contextuality,” Liu Neng (2018) identified four methodological strategies: a pure qualitative space-based sociology; qualitative space-based sociology, such as a pilot study; a qualitative and quantitative space-based sociology with single-level unit analysis; a qualitative and quantitative space-based sociology with multi-level unit analysis. “Spatial contextuality” appears to be a Post-Western concept for the practice of fieldwork in glocal contexts.

The Post-Western methodology is also based on *transnational ethnographies of recognition* and moral economies(Roulleau-Berger 2012). We have considered that both the people we met during our surveys and ourselves have the same bases of competencies at our disposal. In an anti-piratic way (Tilley 2017), we have adopted an approach that rejected “methodological irony,” otherwise known as scholarly knowledge, to produce a concurrent analysis, which sometimes even corrects the attitudes of the members of ordinary society (Watson 2001). For example, we have considered the *nongmingong* (peasant-workers) requests for recognition in China (Roulleau-Berger 2009) and similar requests of the young French-Maghrebi who live in working-class suburbs in French cities (Roulleau-Berger 1999). The definition of the framework of the research experience can thus be developed around the production of moral economies or the transaction, circulation, and exchange of moral and symbolic goods such as confidence, reputation, and consideration. The production of moral economies is the foundation of the interactions between researchers and the individuals they meet in various societal contexts and local situations where sociologists are increasingly confronted with an increase in demands for social and public recognition by populations in situations of vulnerability, poverty, and social or economic disaffiliation. Thus, it is necessary to consider the diversity of multi-situated fields by referring to places of social conflict and requests for recognition.

Producing an ecology of knowledge depends on the competencies of individuals and groups as well as those of the sociologist who constructs them based on honor systems, adjustments, links in meanings given to actions in the research process, and produces moral economies. Here, the production of knowledge imposes negotiated competencies between the sociologist and the actors that will give rise to cooperative knowledge, abilities to exchange and share competencies, and correct and readjust action. More precisely, it is about mastering systems to return knowledge upon which configurations of the actors’ experiences and activities are based and understand the grammar of situations and interactions to which the experiential and pragmatic engagement of the actors conform (Cefaï 2007).

### Post-Western space, common and situated knowledge

In this section, we address the continuities and discontinuities of major theoretical issues in sociology. Although not exhaustive, this approach is rather comprehensive regarding what remains of common and situated knowledge in Post-Western sociology. We have identified the three following topics as illustrations of shared post-western space:

- Migration and integration
- Ethnicity and space
- Ecological change and global risks

#### Migration and integration

The process of urban integration in sociology is often related to the question of migration. First, the assimilation approach characterizing the Chicago School’s immigration studies : Park et al. (1925) described how migrants could use adaptation strategies to be settled in urban enclaves (Li and Roulleau-Berger 2013). They would then usually leave these places, become involved in the competition for space with other social groups, and develop their own social trajectory. Burgess dealt with the concepts of urban integration and segregation. The question of upward social mobility among second-generation migrant communities was broached: the assimilation theory initially showed how the children and grandchildren of migrants gradually acceded to the social statuses of the host society and that this process had an element of irreversibility about it.

In the neighborhoods of European cities, particularly in the working-class suburbs of French cities, spatial, social, and economic segregation have increased over the last 20 years. For example, in 2011, young people of North African origin took more than 11 months to get their first job, compared with 6 months or 7 months for those of French descent, and 24 months on average to access a stable employment contract, compared with 15 months for those of French descent. After 3 years of active life, youths of North African origin occupied more precarious jobs than French descent. For children of immigrants, intergenerational mobility seems to be increasingly less linear and may give rise to “segmented assimilation” (Zhou 1997), which reflects the irregular, reversible, unexpected, multidimensional, and differentiated aspects of urban and economic integration (Roulleau-Berger 1999; Roulleau-Berger and alii 2019).

In China, sociologists are interested in understanding the process of social mobility of Chinese migrant workers (Li 2012, 2013) and new immigrants in big cities, for example, the successive waves of Arab and sub-Saharan traders in Guangzhou. Young Chinese migrants form a new urban underclass and experience new poverty in “urban villages” (Lian 2009) or in new urban areas that appear on the outskirts of megalopolises; some of them are forced to leave the big cities and return to their *laojia* (老家hometown) or to move to other cities. Second, young migrant graduates gain access to skilled jobs in Chinese mega-cities as technicians or executives in private or public Chinese enterprises and international enterprises; however, most of them remain migrants and are in situations of employment and housing insecurity. The majority of these young people do not have an urban *hukou* (household registration), are victims of employment discrimination and social disqualification by local authorities: young graduates with a rural *hukou* cannot easily obtain an urban job. Some of them are forced into informal jobs or unemployment (Roulleau-Berger and Yan 2017).

Chinese sociologists use the concepts of social structures, *guanxi*, and individual strategies (Li and Li 2013) to understand the migratory experience in Chinese cities. For example, they analyze how the less endowed migrants, in terms of social capital and economic and symbolic resources, are subjected to the forces of political and economic measures. They use available networks of trust and community solidarities to find social and spatial anchorage in economic spaces. French sociologists tend to use the concepts of structural processes, individuation, and agency. For example, we have noticed a proliferation of biographical bifurcations in trajectories structured in the conjunction of professional mobility and spatial mobility where migrants rely on individual and collective capabilities to live in the city. Therefore, in this case, we can identify *discontinuous continuities* between Chinese and French sociologies.

#### Ethnicity, space, and religion

In Chinese sociology, just as in European sociology, the deconstruction of the ethnic entity should take societal and historical contexts into account, such as colonialism and nationalism, which produce classifications and fixed moral and social borders. This signifies the deconstruction of ethnic categorizations and classifications in a constructivist approach, interethnic relationships according to Frederic Barth, as well as ethnic boundaries, globalized religions, and transnational spaces. In China, the term “ethnic group” is a foreign concept. Before the 1960s, studies of ethnic groups were rare in the Chinese social sciences. Since the 1970s, the theory of ethnic boundaries conceived by Frederik Barth has had broad repercussions in Chinese academic circles and has been widely cited. Thus, *continuous continuities* have been produced around the theory of ethnic boundaries between Western European and Chinese sociologies.

In France, according to Amselle (1990), ethnicity was a fiction or an illusion produced under the influence of imposed identities in a colonial situation. For Tarrius, and in collaboration with Missaoui, L. (2000), ethnicity is a resource mobilized by entrepreneurs of identities in intergroup struggles. The public affirmation of ethnicity and tensions between history and memory was also founded by the struggles of post-colonial migrants (Boubeker 2003); the children of post-colonial immigration cling to their cultural and religious traditions, especially Islamic ones. Transnational space and transnationalism are strongly linked to local specificities that must be analyzed to understand the plural modes of vernacularization of South Asian Islam in Western Europe and Southern Africa (Sadouni 2019).

In Chinese sociology and anthropology, for Fan (2017), the notions of ethnic group and ethnic boundary have brought an entirely new paradigm, and many things people used to take for granted have now begun to receive serious scrutiny. As a result of Barth’s work, many scholars have had second thoughts on how people were categorized in the ethnic identification campaign and how this categorization has changed ethnic configurations in China; more importantly, many scholars are questioning how national, ethnic, or any other kinds of collective identities have been constructed and reconstructed. In this constructivist approach, for example, Fan (2017) conducted an in-depth field study of the two main Hui groups in the Quanzhou region: the Ding clan and the Guo clan; he described the process of localizing Islam through the activities of the Hui clan and the reappearance of Islamic culture. Mi (2010) also developed the concept of “urban Islamic cultural ecology.”

Following in the footsteps of French scholars, Chinese scholars have come to adhere to a constructivist approach to ethnicity (Fan 2017). They conceptualize the dynamic relationships between ethnicity and cultural identity to study ethnic interactions, the relationships between ethnic groups, and the State and the moral boundaries of ethnic groups (He 2017). Nevertheless, the question of ethnicity and religion could be formulated differently in French and Chinese sociologies. For example, situated concepts can be found in Chinese studies, such as the process of “localizing” Islam or even “urban Islamic cultural ecology” (Mi 2010), showing the broad difference between the weakening of urban traditional Muslim communities and rural Muslim communities. So we also can identify *continuous discontinuities* between Western European and Chinese sociologies.

#### Ecological change and global risks

Environmental sociology began in the 1980s and 1990s with the emergence of environmental problems. In Europe, Anthony Giddens and Ulrich Beck’s reflexive modernization theories inspired the notion of *ecological modernization* (Mol and Spaargaren 2000; Mol and Sonnenfeld 2000) as “the social scientific interpretation of environmental processes at multiple scales in the contemporary world.” More recently, the concept of *environmental justice* has become increasingly central (Nygren 2014), referring to inequalities in the spatial distribution of risks. Certain authors suggest that we should consider the interaction between environmental justice and vulnerability (Walker 2009). It is now necessary to consider the environment and global health issues collectively. As is evident with Covid-19, the epidemic and its fields constitute places of encounter for different ontologies and categories such as the notions of environment, risk, and nature, both human and non-human (Le Marcis et al. 2016). The issue of zoonoses and emerging diseases lies at the heart of global health (Biehl 2016). In transnational health governance, humanitarian interventions are based on a dual imaginary: eschatological (the advent of a future global epidemic) and technological (confidence in the ability of technologies to respond to the risk).

In China, Wang (1999) showed a deterioration of the ecological environment in agriculture in the 1980s. Environmental sociology was introduced as a discipline in China only in the mid-1990s. Hong (2001, 2010) analyzed the social factors of environmental destruction in contemporary China: (1) the transformation of the social structure of industrialization, urbanization, and regional differentiation; (2) the transition of economic and political systems and the market economy; (3) decentralization, reform, and change of control; and (4) new values with moral decline, consumerism, and the intensification of social mobility. Hong (2017) now considers that environmental sociology should provide an understanding of China’s social transformations by considering social subjects’ perceptions of environmental problems, their reactions, institutional arrangements, and changes in cultural values. A body of research has shown how rapid urbanization, industrialization, and economic liberalization—that is, compressed modernity—has led to the emergence of environmental problems (Bao and Chen 2011; Liu and Bao 2019). In an environmental insecurity’s context, citizens today have less and less confidence in governments. More fundamentally, mistrust is one of the great challenges in Chinese society. Zhao and Shi (2016) distinguished types of interpersonal trust, including the trust in acquaintances, strangers, and governments and social institutions. Several researchers (Huang 2009; Jing 2009; Luo 2010) have shown how peasant struggles and resistances are organized to defend environmental rights.

Li and Wang (2016), in a political context of an “ecological civilization construction,” have shown that multi-governance is constructed around ecological migration organized by local and central governments. Jiang (2019) is researching rural environmental governance in combining rural interest, karma, and kinship. Zhang (2019b) considers socioeconomic systems, natural ecosystems, local culture to analyze how institutional arrangements and ecological knowledge are tested and revised in the fabric of adaptive governance and climate change. Zhang (2019a) introduces waste governance and green activism in China and shows how intermediate actors expand a “civic” governing space under the radar of State regulation. Recently, the work of Chen and Xie (2016), starting from the question of the environmental resistance of the peasants, shows that the collective mobilizations in China are built today through processes of influence, diffusion, and diffraction between distinct forms of action that can mobilize new types of intermediate actors.

After the Fukushima accident in Japan, distrust, anxiety, and “protest in the street” characterized Japan’s civil society. Hasegawa (2015) analyzed which resources can be mobilized and under what conditions, and how the interests, values, and beliefs of citizens and social movement activities can be adjusted and brought into alignment.

From a broader perspective, in European sociology, the emergence of “public arenas” (Cefaï 2007; Poulain 2017) raises the issue of their democratic nature, as assessed by the modes of participation and the nature of democratic processes involved in the reconstruction period. The process of emergence of “public arenas” and of different commitment regimes pertains to distinct institutional actors, their forms of mobilization, and coordination to produce “interactional citizenship” (Colomy and Brown 1996).

European, Japanese, and Chinese sociologists are invited to revise the way of conceiving their plurality around social and ecological change. Environmental inequalities and injustices could generate a new perspective for social and spatial justice within a cosmo-political perspective. Reproduction theories and theories of transformation could be combined to understand the complexity of the social construction of risks and disasters by bringing to light *continuous continuities* between Western European and Asian sociologies. If according to Beck (2013), reproduction theories (Atkinson 2007; Bourdieu 1979) produce a “narrative of continuity” concerning the unequal distributions of goods, they miss the cosmopoliticization of the poor, their multi-ethnic, multi-religious, transnational life-forms, and identities. Thus, we are compelled to combine critical sociology and pragmatist sociology (Boltanski 2010) to apprehend the continuities and discontinuities in the reproduction and production of social inequalities in local and global risk societies. Concerning present-day East Asia, Beck (2013) spoke of a cosmopolitan risk community (or “Cosmo-Climate”). He has distinguished “cosmopolitan empathy” and the sub-politics of “cosmopolitanism from below.”

Li Peilin in China, Shujiro Yasawa in Japan, and Chang Kyung-Sup in Korea posit that we cannot conceive the risk issue in the same terms in Europe and in Asia because different “compressed modernities” mean complexities, diversities, and heterogeneities in the risks that people face in East Asia today that overtake those faced by the Western world. Here, we can again speak to the *discontinuous discontinuities* between European and Asian sociologies.

## Conclusion

What do the future of sociology and social sciences look like? Due to Covid-19 pandemic, we must invent a new future for social sciences in an increasingly uncertain world by protecting sociology. According to Connell (2019), we can avoid both an “oligarchic sociology”—which serves the purposes of dominant powers, transnational corporate managers, and financial oligarchs—and a “residual sociology”—in which researchers are relegated by neo-liberal powers to produce a science of the losers while simplified neoclassical economics takes charge of major policy issues. It is a question of promoting democratic sociology to contest Northern hegemony in the global economy of knowledge. Post-Western sociology could be defined as a “knowledge of hospitality.” In this ecology of connectedness, local and indigenous theories become alive, visible, and active, and they are mobilized to produce alternative or heterotopian canons of knowledge. However, we are still taking into account the control of academic knowledge, the disciplinary divides, and gatekeeping within certain European and North American scholarship circles, including the hierarchization of non-Western scholarship and its relegation into area studies (Patel 2019). In fact, epistemic autonomies become plural and diverse, even hierarchical among themselves, without this dynamic of re-composition of the geographies of knowledge in the social sciences being accurately perceived on the side of the Western worlds. The question of Western hegemonies continues to arise through the process of recognition, visibility, and legitimacy of this plurality of epistemic autonomies. Today, with shared, cross-disciplinary, and multi-situated fieldwork practices, we can improve the relationship between knowledge of hospitality and political geography by formulating non-hegemonic sociological perspectives. Furthermore, we can build a “new” ecology of knowledge in *the Western*-*West*, *the non*-*Western*-*West*, *the semi*-*Western West*, *the Western East*, *the Eastern East*, and *the re*-*Easternized East* to understand our common condition in the world.

## References

[CR1] Alatas, Syed Hussein. 1974. The captive mind and creative development. *International Social Science Journal* 24 (4): 691–700.

[CR2] Amselle, J.L. 1990. *Logiques métisses : anthropologie de l’identité en Afrique et ailleurs*. Paris: Payot.

[CR3] Atkinson, W. 2007. Beck, individualization and the death of class: a critique. *British Journal of Sociology* 58 (3): 349–366. 17727498 10.1111/j.1468-4446.2007.00155.x

[CR4] Bao, Zhiming, and Zhanjiang Chen. 2011. Environmental dimension ’s directions and limitations of China experience: review and reflection on studies of environmental sociology in China. *Sociological Studies.* 26 (6): 196–210.

[CR5] Bastide, L. 2015. *Habiter le transnational: Espace, travail et migration entre Java, Kuala Lumpur et Singapour*. Lyon: ENS Éditions.

[CR6] Beck, U. 2000. The cosmopolitan perspective: sociology of the second age of modernity. *The British Journal of Sociology* 51 (1): 79–105.

[CR7] Beck, U. 2013. *Risk, class, crisis, hazards and cosmopolitan solidarity/risk community – conceptual and methodological clarifications. Working Papers Series*. No 3. FMSH.

[CR8] Beck, U., and E. Grande. 2010. Varieties of second modernity : the cosmopolitan turn in social and political theory and research. *The British Journal of Sociology* 61 (3): 409–444. 20857607 10.1111/j.1468-4446.2010.01320.x

[CR9] Bhabha, H. 2007. *The location of culture. A postcolonial theory*. Paris: Payot.

[CR10] Bhambra, G.K. 2014. *Connected sociologies*. London/New York: Bloomsbury.

[CR11] Bhargava, R. 2013. *Pour en finir avec l’injustice épistémique du colonialisme, Socio*, 41–77.

[CR12] Biehl, J. 2016. Theorizing global health. *Medicine Anthropology Theory* 3 (2): 127–142.

[CR13] Blagojevic, M. 2010. The catch 22 syndrome of social scientists in the semiperiphery : exploratory sociological observations. *Sociologica* LII: No. 4.

[CR14] Boatcă, M. 2014. Inequalities unbound: transregional entanglements and the creolization of Europe. In *Postcoloniality-Decoloniality-Black Critique: Joints and Fissures*, ed. S. Broeck and C. Junker, 211–230. Frankfurt and New York: Campus.

[CR15] Boatcă, M. 2015. *Global inequalities beyond occidentalism*. Farnham: Ashgate Publishing.

[CR16] Boltanski, L. 2010. *De la critique. Précis de sociologie de l’émancipation (On critique. A sociology of emancipation)*. Paris: Gallimard.

[CR17] Bonelli, C., and D. Vicherat Mattar. 2017. Towards a sociology of equivocal connections. *Sociology* 51 (1): 60–75.

[CR18] Boubeker, A. 2003. *Les mondes de l'ethnicité : la communauté d'expérience des héritiers de l'immigration maghrébine*. Paris: Balland.

[CR19] Bourdieu, P. 1979. *La distinction. Critique sociale du jugement (Distinction: A social critique of the judgement of taste)*. Paris: Les Éditions de Minuit.

[CR20] Brandel, A., V. Das, and S. Randeria. 2018. Locations and locutions: unravelling the concept of world anthropology. In *Post-Western Sociology-From China to Europe*, ed. L. Roulleau-Berger and Li Peilin. London and New York: Routledge.

[CR21] Cefaï, D. 2007. *Pourquoi se mobilise-t-on ? Les théories de l'action collective*. Paris: La Découverte.

[CR22] Chakrabarty, D. 2000. *Provincializing Europe: Postcolonial thought and historical difference*. Newjersy: Princeton University Press.

[CR23] Chang, Kyung-Sup. 2010. The second modern condition? Compressed modernity as internalized reflexive cosmopolitization. *The British Journal of Sociology* 61 (3): 444–465. 20840427 10.1111/j.1468-4446.2010.01321.x

[CR24] Chang, Kyung-Sup. 2017a. Compressed modernity in South Korea: constitutive dimensions, manifesting units, and historical conditions. In *The Roultegde Handbook of Korean Culture and Society*, ed. Y. Kim, 75–92. New York: Routledge Publishers.

[CR25] Chang, Kyung-Sup. 2017b. China as a complex risk society. *Temporalités* 26.

[CR26] Chen, Guangjin. 2017. Modern transformation of Chinese structures since the Reform. In *The Fabric of Sociological Knowledge: The Exploration of Post-Western Sociology*, ed. Lizhong Xie and L. Roulleau-Berger, 209–252. Beijing: Beijing University Press.

[CR27] Chen, Tao. 2013. Artificial society or natural society Durkheim's critique of social contract theory. *Sociologial Studies* 3: 47–75.

[CR28] Chen, Tao, and Jiabiao Xie. 2016. The mixed resistance: Analysis of environmental struggle by peasants. *Sociological Studies.* 3: 25–46.

[CR29] Cicchelli, V. 2018. Plural and shared : the sociology of a cosmopolitan world. Boston/Leiden: Brill Publishers.

[CR30] Colomy, P.J., and D. Brown. 1996. Goffman and interactional citizenship. *Sociological Perspectives* 39 (3): 371–381.

[CR31] Connell, Raewyn. 2019. *The good university*. London: Zed Books.

[CR32] Dirlik, A. 2007. *Global modernity: modernity in the age of global capitalism*. Paradigm Publisher.

[CR33] Dufoix, S. 2013. Les naissances académiques du global (Global’s academic births). In *Le tournant global des sciences sociales*, ed. A. Caillé and S. Dufoix, 27–44. Paris: La Découverte.

[CR34] Eisenstadt, S. 2000. *Multiple modernities*. New Brunswick: Transaction Publishers.

[CR35] Elliott, E., M. Katagiri, and A. Sawai. 2013. *Japanese social theory: from individualization to globalization in Japan today*. Oxon/New York: Routledge.

[CR36] Fan, Ke. 2017. Barth’s ethnic boundary and the understanding of frontier in Chinese context. *Academic Monthly* 49 (7).

[CR37] Fei, Xiaotong. 1992. *From the soil: the foundation of Chinese society.*. Berkeley: University of California Press.

[CR38] Han, S.-J., and Y.-H. Shim. 2010. Redefining second modernity for East Asia: a critical assessment. *The British Journal of Sociology* 61 (3): 465–488. 20840428 10.1111/j.1468-4446.2010.01322.x

[CR39] Han, Sang-Jin. 2019. *Confucianism and reflexive modernity:bringing community back to human rights in the age of global risk society*. Leiden/Boston: Brill Publishers.

[CR40] Hasegawa, K. 2015. *Beyond Fukushima: toward a post-nuclear society*. Melbourne: Transpacific Press.

[CR41] Hasegawa, K. 2018. Continuities and discontinuities of Japan's political activism before and after the Fukushima nuclear accident. In *Social Movements and Political Activism in Contemporary Japan: Re-emerging from Invisibility*, ed. David Chiavacci and Julia Obinger, 115–135. Oxford, Routledge.

[CR42] He, Hu. 2017. On the construction of ethnic groups and cultural identity. *Journal of Dalian University for Nationalities* 19 (4): 239–299.

[CR43] He, Rong. 2020. Max Weber’s view on China: an investigation based on research position and text source. *New Perspectives on World Literature* (3):49–59.

[CR44] He, Yijin. 2018a. An alternative autonomy: the self-adaptations of Chinese sociology in the 1950s. In *Post-Western Sociology: from China to Europe*, ed. L. Roulleau-Berger and Li Peilin. Oxon/New York: Routledge.

[CR45] He, Yijin. 2018b. Variations of the Other: the context and fluctuations of sinicizing sociology in China. *Sociological Review of China* 6 (6): 84–95.

[CR46] Hong, Dayong. 2001. *Social changes and environmental issues: sociological interpretation of environmental issues in contemporary China*. Beijing: Capital Normal University Publishing House.

[CR47] Hong, Dayong. 2010. Theoretical awareness and development of environmental sociology in China. *Journal of Jilin University* 50 (3): 109–116.

[CR48] Hong, Dayong. 2017. Environmental sociology: objects, theories and values. *Thinking* 43 (1): 78–92.

[CR49] Huang, Jialiang. 2009. Environmental rights protection through group litigation: multiple dilemmas and action logic-Based on the analysis of an environmental lawsuit in P County, South China. *Rural China* 6: 183–220.

[CR50] Jiang, Pei. 2019. The social foundation of rural environemental governance- A case study based on the classification of garbage in Lujia Village, Zhejiang Province Communication. In *Workshop LIA CNRS-ENS Lyon /CASS 2019, June 17-20*^*th*^*, Compressed modernities, ecological risks and disasters in Europe, Asia, Latin America, Africa*.

[CR51] Jing, Jun. 2009. Cognition and consciousness: environmental struggle in a Northwest village. *Journal of China Agricultural University (Social Science Edition)* 26 (4): 5–14.

[CR52] Kang, Jung-In. 2006. Academic dependency: Western –centrism in Korean political science. *Korean Journal*: 115–135.

[CR53] Katagiri, M. 2013. The three selves in Japanese society: individualized, privatized, and psychological selves in Japanese Social Theory. In *Japanese Social Theory. From individualization to globalization in Japan today*, ed. E. Elliott, M. Katagiri, and A. Sawai, 139–158. Oxon/New York: Routledge.

[CR54] Kilani, M. 2009. *Anthropologie. Du local au global*. Paris: Armand Colin.

[CR55] Kim, Seung Kuk. 2014. East Asian communauty as hybridization : a quest for East Asianism. In *A Quest for East Asian Sociologies*, ed. Kim Seung Kuk, Li Peilin, and Shujiro Yasawa. Seoul: Seoul University Press.

[CR56] Kim, Seung Kuk, Li Peilin, and Yasawa Shujiro, eds. 2014. *A quest of East Asian sociologies*, Seoul: Seoul National University Press.

[CR57] Koleva, S. 2018. *Totalitarian experience and knowledge production sociology in Central and Eastern Europe 1945-1989*. Leiden/Bostonn: Brill Publishers.

[CR58] Kwang-Yeong, Shin. 2013. The emergence of hegemonic social sciences and strategies of non (counter) hegemonic social sciences. In *Theories About Strategies Against Hegemonic Social Sciences*, ed. M. Kuhn and S. Yasawa, 77–94. Tokyo: Seijo University.

[CR59] Le Marcis, F., with Brives, C. & Sanabria, E., 2016. What’s in a context? tenses and tensions in evidence-based medicine. *Medical Anthropology*, 35(5) : 369-376. 10.1080/01459740.2016.1160089 27618221

[CR60] Li, Chunling. 2013. Institutional and non-institutional path: Different processes of socio-economic status attainment of migrants and non-migrants in China. In *China’s Internal and International Migration*, ed. Peilin Li and Roulleau-Berger, 26–40. L. Oxon/New York: Routledge Publishers.

[CR61] Li Lulu. 2008. Transition and socialstratification in Chinesecities. In *New Chinese sociology*, ed. Roulleau-Berger, L., Guo, Yuhua, Li, Peilin, Liu, Shiding, 1: 19–145. Paris: Editions du CNRS.

[CR62] Li, P, Jingdong Qu, and Yabin Yang. 2009. *An introduction to Chinese sociological classics*. Peking: Social Sciences Academic Press.

[CR63] Li, Peilin. 2012. Chinese society and the China experience. In *Chinese society-Change and transformation*, ed. Li Peilin. London/ New York: Routledge.

[CR64] Li, Peilin. 2015. La modernisation orientale et l’expérience chinoise. *Socio* 5: 25–45.

[CR65] Li, Peilin, Qiang Li, and Rong Ma. 2008. *Sociology and Chinese society*. Beijing: Academic Press of Social Sciences.

[CR66] Li, Peilin, and Wei Li. 2013. The work situation and social attitudes of migrant workers in China under the crisis. In *China’s internal and International Migration*, ed. Peilin Li and L. Roulleau-Berger, 3–26. Oxon/New York: Routledge Publishers.

[CR67] Li, Peilin, and Jingdong Qu. 2011. *History of sociology in China in the first half of the twentieth century*. Beijing: Social Sciences Academic Press.

[CR68] Li, Peilin, and Jingdong Qu. 2016. *La sociologie chinoise avant la Révolution*. Paris: Editions FMSH.

[CR69] Li, Peilin, and L. Roulleau-Berger, eds. 2013. *China’s internal and international migration*. London/ New York: Routledge Publishers.

[CR70] Li, Peilin, and Xiaoyi Wang. 2016. Reducing double risks in ecological degradation and poverty : a research on ecological migration in the Ningxia Hui autonomous region of China. In *Ecological risks and disasters -New experiences in China and Europe*, ed. Li Peilin and L. Roulleau-Berger. Oxon/New York: Routledge.

[CR71] Lian, Si. 2009. *Yizu*. Guilin: Guangxi Normal University Press.

[CR72] Liu, Min, and Zhiming Bao. 2019. “Compressed modernization” in Western ethnic areas and its ecological environment issues-the case of Alashan, Inner Mongolia. *Social Sciences in Yunnan* 2: 113–119.

[CR73] Liu, Neng. 2008. Field research methodology reconsidered: reading Earthbound China’s Introduction. In *Fei Hisao-Tung and China’s sociology and anthropology*, ed. Rong Ma, Shiding Liu, Zeqi Qiu, and Naigu Pan, 789–798. Beijing: Social Sciences Academic Press.

[CR74] Liu, Neng, and Su, Wu. 2019. On the indigeneization of sociology as an academic movement. *Journal of University of Jinan* 29 (1): 5–16.

[CR75] Liu, W., and Yalin Wang. 2017. The re-understanding of connotation of sociological indigenization in China. *Journal of Social Sciences* 19 (1): 50–59.

[CR76] Luo, Yajuan. 2010. Environmental resistance in rural industrial pollution: a case study of Dongjing Village. *Academia Bimestris* 2: 91–97.

[CR77] Madan, T.T.N. 2011. *Sociological traditions, methods and perspectives in the Sociology of India*. New Delji: Sage.

[CR78] Mbembe, A. 2006. Qu’est-ce que la pensée postcoloniale? *Esprit* 330: 117–133.

[CR79] Mbembe, A. 2018. *Politiques de l’inimitié*. Paris: Payot.

[CR80] Mi, Shoujiang. 2010. The process of urbanization of Islam in China and its development trend. *The Religious Cultures in the World* 1.

[CR81] Mol, A.P.J., and D.A. Sonnenfeld, eds. 2000. *Ecological modernization around the world. Perspectives and critical debates*. London and Portland: Frank Cass/Routledge.

[CR82] Mol, A.P.J., and G. Spaargaren. 2000. Ecological modernization and consumption : a reply. *Society and Natural Resources* 17: 261–265.

[CR83] Neng, Liu. 2018. Returning to space-based sociology: inheriting Professor Fei Hsiao-Tung’s academic heritage. In *Post-Western sociology-From China to Europe*, ed. L. Roulleau-Berger and Peilin Li. London and New York: Routledge.

[CR84] Nishihara, K. 2013. Phenomenological sociology in Japan and its significance for contemporary social research. In *Japanese social theory. From individualization to globalization in Japan today*, ed. A. Elliott, M. Katagiri, and A. Sawai, 20–36. London/New York: Routledge.

[CR85] Nomiya, K. 2019. Transforming the ominous into happiness: how antinuclear drive was tamed in the post-war Japan? In *Issues and Themes in Contemporary Society. Essays in Honour of Professor Ishwar Modi*, ed. B.K. Nagla and V.K. Srivastava, 14–26. New Delhi: Rawat Publications.

[CR86] Nygren, A. 2014. Eco-Imperialism and environmental justice. In *Routledge International Handbook of Social and Environmental Change*, ed. S. Lockie, D.A. Sonnenfeld, and D.R. Fisher, 58–70. London and New York: Routledge.

[CR87] Pan, Quentin. 1931. *The family problem in China*. Xinyue: Bookstore.

[CR88] Park, Myoung-Kyu, and Kyung-Sup Chang. 1999. Sociology between Western theory and Korean reality : accomodation, tension and a search of alternatives. *International Sociology* 14: 139 10.1177/0268580999014002002:139-156.

[CR89] Park, R.E., E.W. Burgess, and R.D. McKenzie. 1925. *The city: suggestions for investigations of human behavior in the urban environnment*. Chicago: University of Chicago Press.

[CR90] Patel, S. 2010. At crossroads. Sociology in India. In *The ISA Handbook of diverse sociological tradition*, ed. Patel. London: Sage Publishers.

[CR91] Patel, S. 2017. Colonial modernity and methodological nationalism : the structuring of sociological traditions in India. *Sociological Bulletin* 66 (2): 125–144.

[CR92] Patel, S. 2019. Gazing forward through the Prism of the Colonial Past: Notes on sociology’s contradictory histories in India. Communication at Seminar. *Toward a Non-Hegemonic World Sociology* Saint-Emilion 2019 June the 25-28.

[CR93] Pleyers, G. 2011. *Alter-Globalization*. Cambridge: Polity Press.

[CR94] Poulain, J.P. 2017. Socio-anthropologie du « fait alimentaire » ou *food studies.* Les deux chemins d’une thématisation scientifique. *L'Année Sociologique* 1 (67): 23–46.

[CR95] Qu, J. 2017a. Back to historical views, reconstructing the sociological imagination : the new tradition of classical and historical studies in the modern Chinese transformation. *Chinese Journal of Sociology* 3: 135–166.

[CR96] Qu, Jingdong. 2017b. After sacred society: to commemorating 100th anniversary of Emile Durkheim’s death. *Chinese Journal of Sociology* 4.

[CR97] Raman, R.K. 2017. Subaltern modernity : Keral, the Eastern theatre of resistance in the global South. *Sociology* 51 (1): 91–110.

[CR98] Roulleau-Berger L, 1999, *Le travail en friche. Les mondes de la petite production urbaine,* La Tour D'aigues, Editions de l'Aube.

[CR99] Roulleau-Berger, L. 2008. Pluralité et identité de la sociologie chinoise. In *La nouvelle sociologie chinoise*, ed. L. Roulleau-Berger, Guo Yuhua, Li Peilin, and Liu Shiding. Paris: CNRS Publishers.

[CR100] Roulleau-Berger, L. 2009. Circulation, disqualification, autonomie des migrants en Chine continentale. *Espaces, Populations et Sociétés* no 3: 419–438.

[CR101] Roulleau-Berger, L. 2011. *Désoccidentalisation de la sociologie, L’Europe au miroir de la Chine*. La Tour d’Aigues: Editions de L’aube. Translated in Chinese by Social Sciences Academic Press, 2014.

[CR102] Roulleau-Berger, L. 2012. *Sociologies et cosmopolitisme méthodologique*. Toulouse: PUM.

[CR103] Roulleau-Berger, L. 2015. Sciences sociales post-occidentales : de l’Asie à l’Europe. *Socio* 5: 9–17.

[CR104] Roulleau-Berger, L. 2016. *Post-Western revolution in sociology. From China to Europe*. Boston/Leiden: Brill Publishers.

[CR105] Roulleau-Berger, L., et al. 2019. *Young migrants and descendants of migrants, work and urban skills in Lyon*. Milan and Shanghai: Research Report CNRS-INJEP/CMIRA.

[CR106] Roulleau-Berger, L., Yuhua Guo, Peilin Li, and Shiding Liu, eds. 2008. *La Nouvelle Sociologie chinoise (New Chinese sociology)*. Paris: Editions du CNRS.

[CR107] Roulleau-Berger, L., and Peilin Li, eds. 2012. *European and Chinese sociologies. A new dialogue*. Boston/Leiden: Brill Publishers.

[CR108] Roulleau-Berger, L., and Peilin Li, eds. 2018. *Post-Western sociology. From China to Europe*. Leiden: Brill Publishers.

[CR109] Roulleau-Berger, L., and Zhengai Liu. 2012. La théorie de la religion chez Durkheim et la sociologie chinoise. *Archives de Sciences Sociales des Religions.* 159: 135–151.

[CR110] Roulleau-Berger, L., and J. Yan. 2017. *Travail et migration: Jeunesses chinoises à Shanghai et Paris*. La Tour d’Aigues: L’Aube.

[CR111] Sadouni, S. 2019. *Muslims in Southern Africa : Johannesburg’s Somali diaspora*. London: Palgrave Macmillan.

[CR112] Saeki, K. 1997. *Who is a citizen?* Tokyo: PHP.

[CR113] Saïd, E.W. 2003. *L’orientalisme*. Paris: Seuil.

[CR114] Santos, B.S. 2014. *Epistemologies of the South: justice against epistemicide*. New York: Routledge.

[CR115] Santos, B.S., and G.K. Bhambra. 2017. Introduction: global challenges for sociology. *Sociology* 51 (1): 3–10.

[CR116] Savransky, M. 2017. A decolonial imagination: sociology, anthropology and the politics of reality. *Sociology* 51 (1): 91–110.

[CR117] Shen, Y., and Yuhua Guo. 2010. A new agenda for the sociology of transformation in China. In *The ISA Handbook of diverse sociological tradition*, ed. S. Patel, 305–313. London: Sage Publishers.

[CR118] Sun, Benwen. 1935. *Principles of sociology (Shehuixue yuanli)*. Beijing: Shangwu yinshuguan.

[CR119] Sun, Feiyu. 2013. *Social suffering, and political confession*. Singapore: World Scientific.

[CR120] Sun, Feiyu. 2018. *Methodology and Life World*. Beijing: Xinzhi Sanlian Bookstore.

[CR121] Takakura, H. 2016. Lessons from anthropological projects related to the great East Japan earthquake and tsunami : intangible culture heritage survey and disaster salvage anthropology. In *World Anthropologies in Practice*, ed. J. Gledhill. London/New York: Bloomsbury.

[CR122] Tarrius, A., and in collaboration with Missaoui, L. 2000. *New cosmopolitanisms: mobilities, identities, territories*. La Tour d’Aigues: l’Aube.

[CR123] Therborn, Göran. 2003. Entangled modernities. *European Journal of Social Theory* 6 (3): 293–305 10.1177/13684310030063002.

[CR124] Tilley, L. 2017. Resisting piratic method by doing research otherwise. *Sociology* 51 (1): 27–42.

[CR125] Walker, G. 2009. Beyond distribution and proximity : exploring the multiple spatialities of environmental justice. *Antipode.* 41 (4): 614–636.

[CR126] Wang, Hejian. 2005. Visit Durkheim again: the social construction of morality in modern economy. *Sociological Studies* 1: 149–247.

[CR127] Wang, Yuesheng. 1999. Family responsibility system, farmer behavior and environmental and ecological issues in agriculture. *Journal of Peking University* 3.

[CR128] Watson, R. 2001. Continuité et transformation de l’ethnométhodologie. In *L’ethnométhodologie. Une sciences radicale*, ed. M. De Fornel, A. Ogien, and L. Quéré, 15–29. Paris: La découverte.

[CR129] Wen, Jun, and Yan Wang. 2012. Sun Benwen and sinicization of sociology. *Journal of Social Sciences* 14 (5): 37–43.

[CR130] Xie, Lizhong. 2012a. *Postsociology*. Beijing: Social Sciences Academic Press.

[CR131] Xie, Lizhong. 2012b. *The Discursive construction of social reality: analyzing the new deal for example*. Beijing: Peking University Press.

[CR132] Xie, Lizhong. 2020. Indigenization, internationalization, construction of academic sociology and Chinese singularity: from geographic pluralism to discourse pluralism. *Sociological Research* 35 (1): 1–15.

[CR133] Xie, Lizhong, and L. Roulleau-Berger, eds. 2017. *The fabric of sociological knowledge*. Beijing: Peking University Press.

[CR134] Xie, Yu. 2018. Going beyond the misunderstandings in the discussion of the indigenization of Chinese sociology. *Sociological Research* 2: 1–13.

[CR135] Yama, Y. 2012. What is symbolic recovery. *Japanese Studies* 18: 63–102.

[CR136] Yamazaki, M. 1984. *Yawarakai Kojinshugi no Tanjo (The Birth of Soft Individualism)*. Tokyo: Chuo-Koron.

[CR137] Yang, Kaidao. 1930. *Rural autonomy*. Shanghai: WJ Bookstore.

[CR138] Yang, Kun. 1932a. History of French sociology (Faguo shehuixue shilue). *Biance zhoukan* 2(7-8): 106–203.

[CR139] Yang, Kun. 1932b. Schools and tendancies in Chinese modern sociology (Zhongguo xiandai shehuixue zhi paibie yu qushi). *Biance zhoukan* 1(4).

[CR140] Yasawa, S. 2014. Civilizational encounter, cultural translation and social reflexivity : a note on history of sociology in Japan. In *A quest of East Asian Sociologies*, ed. Seung Kuk Kim, Li Peilin, and Shujiro Yasawa, 131–167. Seoul: Seoul National University Press.

[CR141] Yasawa, S. 2019. Toward a non-hegemonic social science, seminar. *Toward a Non-hegemonic World Sociology* Saint-Emilion (France) 2019 June the 25-27.

[CR142] Yatabe, K. 2015. Le dépassement de la modernité japonaise. *Socio* 5: 115–138.

[CR143] Zhai, Xuewei. 2020. On the construction of confucian social theory—dual generation and its propositions. *Sociological Studies* 35 (1): 56–80.

[CR144] Zhang, J. 2019a. Waste crisis in contemporary China : green governance, social activism and technological controversies, Communication. In *Workshop LIA CNRS-ENS Lyon/CASS, Compressed modernities, ecological risks and disasters in Europe, Asia, Latin America, Africa, 2019, June 17-20*^*th*^.

[CR145] Zhang, Qian. 2019b. *Perspectives on sociology of environment*. Beijing: China Social Sciences Press.

[CR146] Zhao, Liwei. 2014. Suicide and the situation of modern people Durkheim’s “suicide typology” and its human foundation. *The Journal of Chinese Sociology* 34 (6): 114–139.

[CR147] Zhao, Yandong, and Changhui Shi. 2016. The structure and change of social trust during post-disaster reconstruction: an example of the Wenchuan earthquake-affected population. In *Ecological risks and disasters -New experiences in China and Europe*, ed. Li Peilin and L. Roulleau-Berger, 13–24. Oxon/New York: Routledge.

[CR148] Zhou, Feizhou. 2018. Action ethics and “relationship society”: the path of sociology sinicization. *Sociological Studies* 1: 41–62.

[CR149] Zhou, Min. 1997. Segmented assimilation: issues, controversies and recent resarch on the new second generation. *International Migration Review* 31 (4): 825–858. 12293212

[CR150] Zhou, Xiaohong. 2004. *Contribution and limitation of Chicago School*. Chengdu: Social Sciences Research.

[CR151] Zhou, Xiaohong. 2020. The indigenization of sociology: narrow or broad? pseudo-problem or true reality? - a discussion with Professor Xie Yu and Professor Zhai Xuewei. *Sociological Studies* 1: 16–36.

